# The fabrication of large-scale sub-10-nm core-shell silicon nanowire arrays

**DOI:** 10.1186/1556-276X-8-405

**Published:** 2013-10-01

**Authors:** Shiming Su, Linhan Lin, Zhengcao Li, Jiayou Feng, Zhengjun Zhang

**Affiliations:** 1 School of Materials Science and Engineering, Key Laboratory of Advanced Materials, Tsinghua University, Beijing 100084, People’s Republic of China

**Keywords:** Core-shell silicon nanowire, Polystyrene sphere, Metal catalytic etching, Self-limiting oxidation, 81.07.Vb, 81.16.Dn, 81.16.He

## Abstract

A combination of template-assisted metal catalytic etching and self-limiting oxidation has been successfully implemented to yield core-shell silicon nanowire arrays with inner diameter down to sub-10 nm. The diameter of the polystyrene spheres after reactive ion etching and the thickness of the deposited Ag film are both crucial for the removal of the polystyrene spheres. The mean diameter of the reactive ion-etched spheres, the holes on the Ag film, and the nanowires after metal catalytic etching exhibit an increasing trend during the synthesis process. Two-step dry oxidation and post-chemical etching were employed to reduce the diameter of the silicon nanowires to approximately 50 nm. A self-limiting effect was induced by further oxidation at lower temperatures (750°C ~ 850°C), and core-shell silicon nanowire arrays with controllable diameter were obtained.

## Background

Silicon is one of the most important semiconductor materials due to its crucial role in modern integrated circuit technology. However, the indirect bandgap structure restricts its future application in optoelectronics. Nowadays, silicon nanomaterials are regarded as promising candidates in various areas such as renewable energy [1-4], biological applications [5,6], and chemical sensors [7-10]. It is also considered that silicon nanostructure, with diameter below the Bohr radius of silicon (4.3 nm), could conquer the physical disability of poor luminescence in bulk Si [11,12]. Several silicon nanostructures, such as porous Si [13-15] and Si nanocrystals [16-18], have been widely studied in the past 20 years. However, little attention has been paid to the luminescence property of silicon nanowires (SiNWs) due to the difficulty of preparing nanowires with the diameter of several nanometers. It has been reported that vapor–liquid-solid (VLS) process is available for the achievement of nanoscale SiNWs [19,20]. Yet, the luminescence stability is poor due to the surface termination conditions. In addition, it is difficult to avoid the creation of defects in the nanowires. Another typical method is a combination of electron beam lithography (EBL), reactive ion etching (RIE), and self-limiting thermal oxidation to fabricate sub-10-nm SiNWs [21-24]. It should be noted that this technique is expensive, and the aspect ratio is highly restricted.

In this paper, we demonstrate a technique based on a combination of template-assisted metal catalytic etching [25-28] and self-limiting oxidation to prepare large-scale core-shell SiNW arrays with an aspect ratio of more than 200:1 and the inner diameter of sub-10 nm. A careful discussion of the morphology and structure of the core-shell SiNW arrays is also included.

## Methods

**Figure 1:**
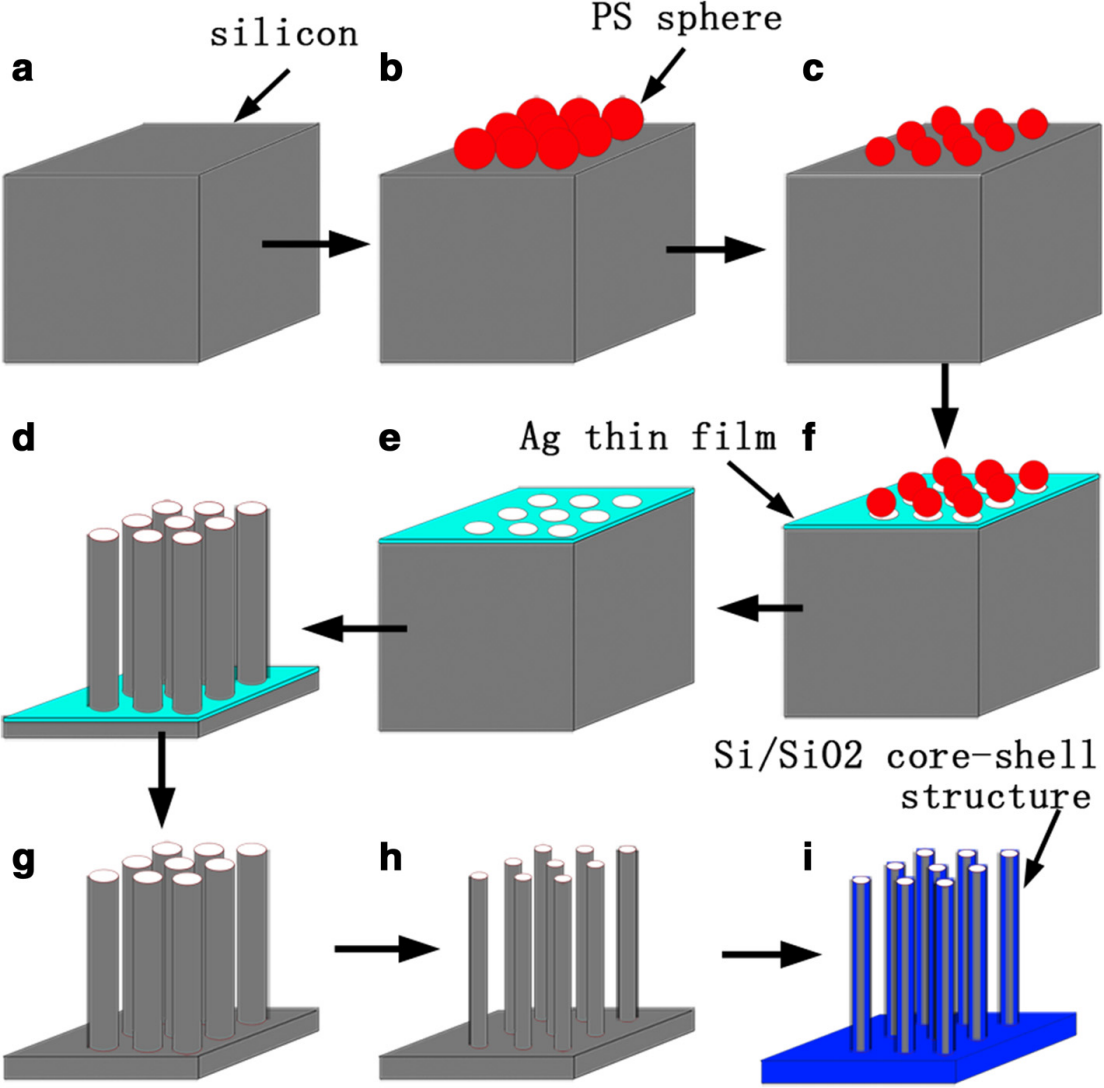
**Schematic depiction of the fabrication process. (a)** Pretreated silicon wafer, **(b)** assembly of PS sphere arrays, **(c)** RIE of the PS spheres, **(d)** deposition of the Ag film, **(e)** removal of the PS spheres, **(f)** metal catalytic etching, **(g)** removal of the residual silver, **(h)** two-step dry oxidation, and **(i)** self-limiting oxidation.

The p-type Si (100) wafers (*ρ* 15 to 20 Ω cm) were cut into 3 cm × 3 cm pieces, degreased by ultrasonic cleaning in acetone, ethanol, and deionized water, and subjected to boiling Piranha solution (4:1 (*v*/*v*) H_2_SO_4_/H_2_O_2_) for 1 h. The overall fabrication process is schematically depicted in Figure 1. The polystyrene (PS) sphere (*D* = 250 nm) solution (10 wt%) was purchased from Bangs Laboratories, Inc. (Fishers, IN, USA). The solution was diluted with deionized water to the concentration of 0.3 wt% and then mixed with ethanol (1:1 (*v*/*v*)). The mixture was ultrasonicated for 30 min to ensure the uniform dispersing of the PS spheres. The 2 cm × 2 cm glass slide used to assist the assembly of the PS sphere template was made hydrophilic through ultrasonication in acetone, ethanol, and deionized water, and then in the Piranha solution for 1 h.

**Figure 2:**
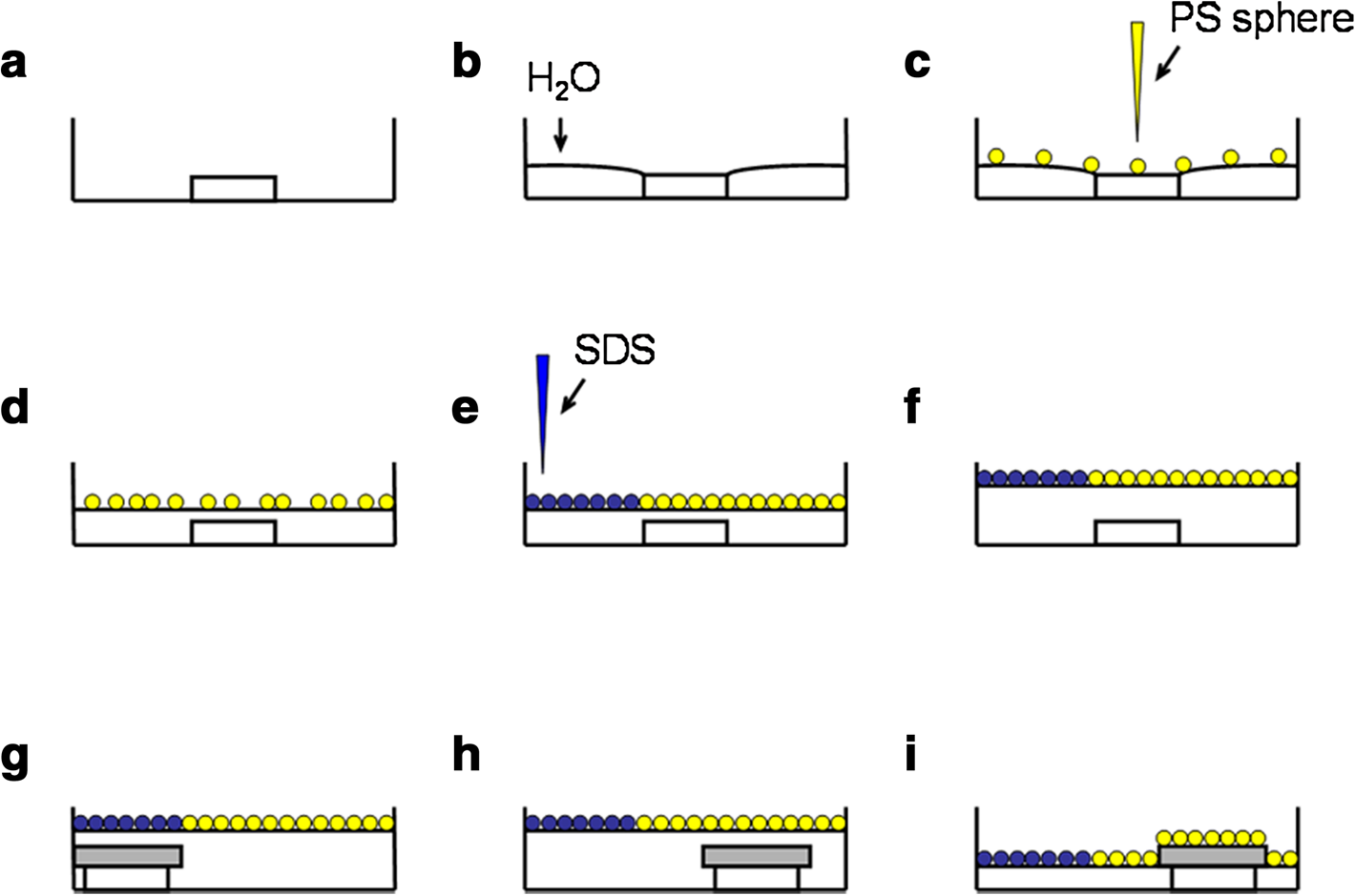
**Schematic depiction of the assembly of monolayer PS sphere arrays. (a)** Pretreated glass in the center of the petri dish, **(b)** adding water, **(c)** adding PS sphere mixture, **(d)** waiting for the water to immerse the glass, **(e)** adding surfactant, **(f)** elevating the water surface, **(g)** pulling the glass to the edge of the petri dish and putting a piece of silicon wafer on it, **(h)** pushing the glass and silicon wafer to the PS sphere side altogether, and **(i)** withdrawing the excess water.

The preparation procedure used to assemble the monolayer PS sphere arrays is illustrated in Figure 2. The pretreated glass slide was placed in the center of a petri dish (*D* = 15 cm), and deionized water was added until the water level was slightly higher than the glass slide's upper surface but did not immerse it. The height difference between the glass and water surface made possible the follow-up self-assembly of the PS spheres on the water. Subsequently, 1,000-μL PS sphere mixture was introduced dropwise on the glass slide, and the PS spheres spread out onto the surface of the water, forming an incompact monolayer. Several droplets of sodium dodecyl sulfate (SDS) solution (1 wt%) were then added, and a compact PS monolayer formed. After elevating the water level and pulling the glass slide to the SDS side using an elbow tweezers, a piece of pretreated silicon substrate was placed on it. Then, they were pushed together to the PS sphere side. The monolayer template could be transferred onto the Si substrate by withdrawing the excess water. Upon the completion of water evaporation, a large-area close-packed monolayer of the PS spheres was formed on the substrate.

The diameter of the PS spheres was reduced via RIE, with an O_2_ flow rate of 40 sccm, pressure of 2 Pa, and applied radio frequency power of 50 W. Ag was sputtered onto the Si substrate, forming a porous Ag film as catalyzer. The PS sphere template was removed from the substrate by ultrasonication in ethanol. The porous Ag film-coated Si substrate was etched in the solution containing deionized water, HF, and H_2_O_2_ at 30°C. The concentrations of HF and H_2_O_2_ were 4.8 and 0.3 M, respectively. The retained Ag film was dissolved with nitric acid (1:1 (*v*/*v*) HNO_3_/H_2_O) for 5 min. The diameter of the as-prepared SiNWs was reduced by dry oxidation in a tube furnace at 1,050°C and post-chemical treatment to remove the oxide layer in the HF solution. At last, the SiNWs, with diameter around 50 nm, were oxidized at 800°C for 10 h. Due to the self-limiting effect, a core-shell structure with sub-10-nm single crystal SiNW was obtained.

The morphology of the SiNW arrays was analyzed using thermally assisted field-emission scanning electron microscope (FE-SEM, JEOL-JSM 7001F, Tokyo, Japan). Transmission electron microscopy (TEM, JEOL-JSM 2011) was further introduced to investigate the core-shell structure.

## Results and discussion

**Figure 3:**
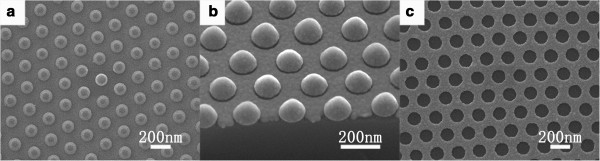
**SEM images describing the formation of the porous Ag film template. (a)** SEM image of the sample after RIE treatment of 55 s. **(b)** SEM image of the sample after 5-min Ag deposition. **(c)** The sample after removal of the PS spheres by ultrasonication.

In the RIE step, the sphere diameter was reduced gradually when the etching time increased, about 176, 141, and 103 nm after RIE of 50, 55, and 60 s, respectively [29]. Figure 3a shows the top-viewed SEM image of the PS spheres with RIE of 55 s. After RIE treatment, the spaces between the nanospheres could be utilized for the subsequent Ag film deposition. Five minutes of deposition can form continuous Ag film with the thickness of around 35 nm, as shown in Figure 3b. The removal of the PS template was carried out, and a porous Ag film, with regularly distributed nanopores (Figure 3c), was available for chemical etching to obtain the SiNW arrays. It should be noted that the diameter of the PS spheres after RIE treatment, the spaces between the PS spheres, and the thickness of the Ag film deposited are important for the removal of the sphere template and the following chemical etching. On one hand, for PS spheres with certain diameter, the Ag film should be thin enough to avoid the conglutination between the PS spheres and the Ag film, which would prevent the removal of the PS spheres. On the other hand, in order to avoid the Ag film from becoming discontinuous, the thickness of the Ag film could not be too thin. It is known that the discontinuous Ag film, or Ag nanoparticles, could also act as catalyzer but yield SiNWs with uncontrollable and non-uniform diameters, which are undesirable in our study. Because ultrasonication was employed here to remove the PS spheres, the width of the porous Ag film should also be considered. Once the width is too small, the film would be destroyed after ultrasonication treatment. Therefore, the spaces between the adjacent PS spheres, which determine the width of the porous Ag film, should not be too limited.

**Figure 4:**
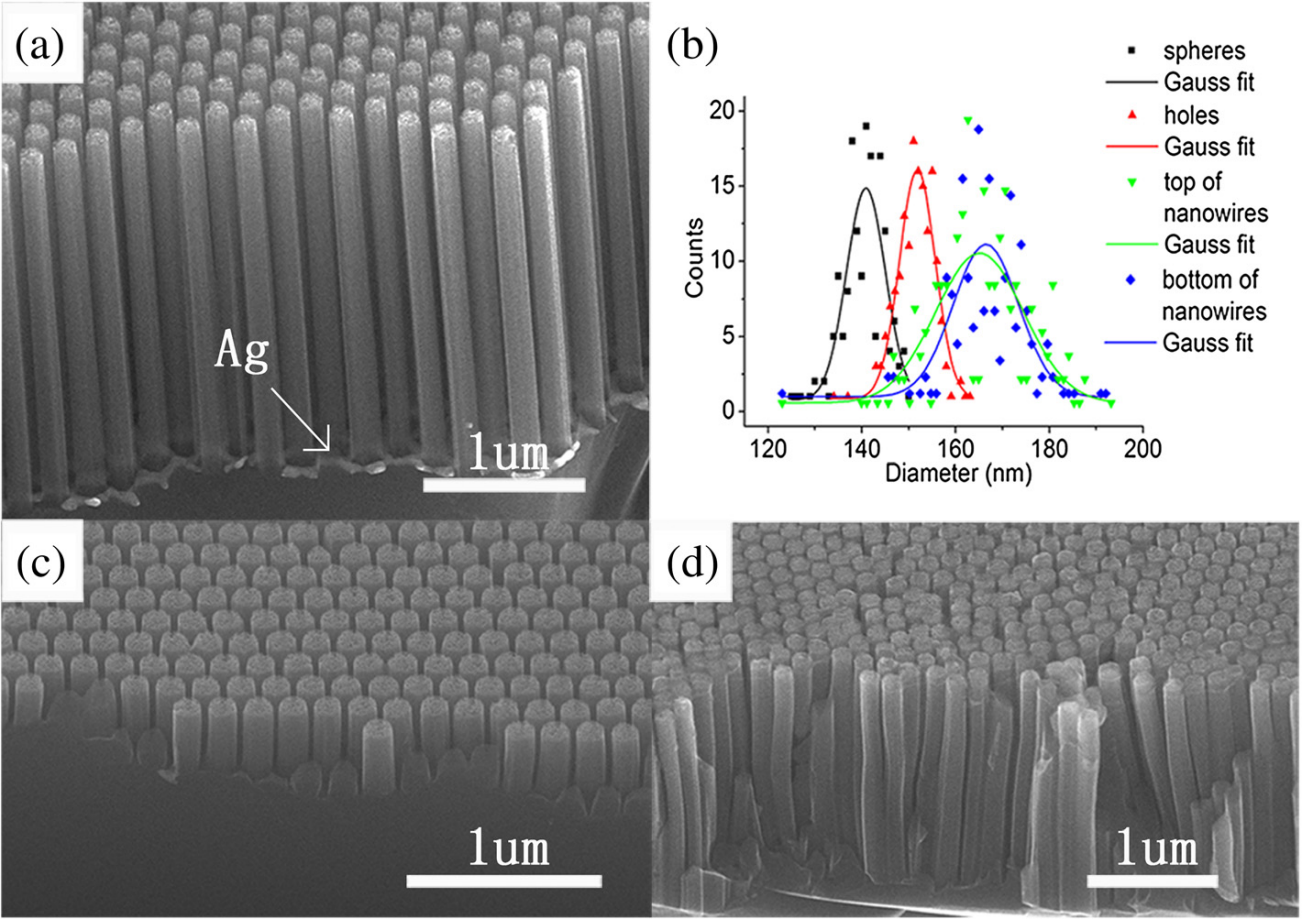
**SEM images of samples after the metal catalytic etching. (a)** SEM image of SiNW arrays after 5-min solution etching. **(b)** Gauss fit of the dimension of the spheres, holes, and top and bottom of nanowires. **(c)**, **(d)** SEM images of samples using 200-nm PS sphere template; the samples have been etched by solution for 2 and 5 min, respectively.

Figure 4a is a typical cross-sectional SEM image of the homogeneously distributed SiNW arrays. The residual Ag thin film at the root of the nanowires explicitly confirms the vertical sinking of Ag during the solution etching process. The size distribution of the diameter reduced PS spheres, the holes on the Ag film, and the top and bottom of the SiNWs has been summarized in Figure 4b. The mean diameter of the spheres, holes, and the top and bottom of the nanowires is 141, 151, 155, and 174 nm, respectively, showing an obvious increasing trend. The silver coated on the PS spheres could increase their diameter and, therefore, cause the size increase of the nanoholes formed on the Ag film. The irregular edges of the holes on the Ag thin film which would locally impede the metal catalytic solution etching might lead to diameter discrepancy between the holes and top of the nanowires. The increase of the dimension from top to bottom of the nanowires might result from the depletion of Ag as the solution etching went on.

The initial diameter of the PS spheres is also crucial for the chemical etching process. Excessive reduction of the sphere size by RIE would prevent the removal of the spheres and the metal catalytic etching. Decreasing the RIE time could avoid excessive reduction of the sphere diameter. However, the gap between the etched spheres would also be limited, leading to the size reduction of the porous Ag film. Figure 4c,d displays the morphology of the SiNW arrays employing PS spheres of 200 nm as the template. At the initial stage of the chemical etching, it is shown that the nanopillars are separated from each other. As the reaction proceeded, the slight dissolution of silver would gradually reduce the size of the porous Ag film, resulting in the increase of the nanowire dimension and, therefore, causing the root section of the nanowires to be connected. Thus, the PS spheres with initial mean diameter of 250 nm are the smallest commercially available spheres that can be used in this experiment.

**Figure 5:**
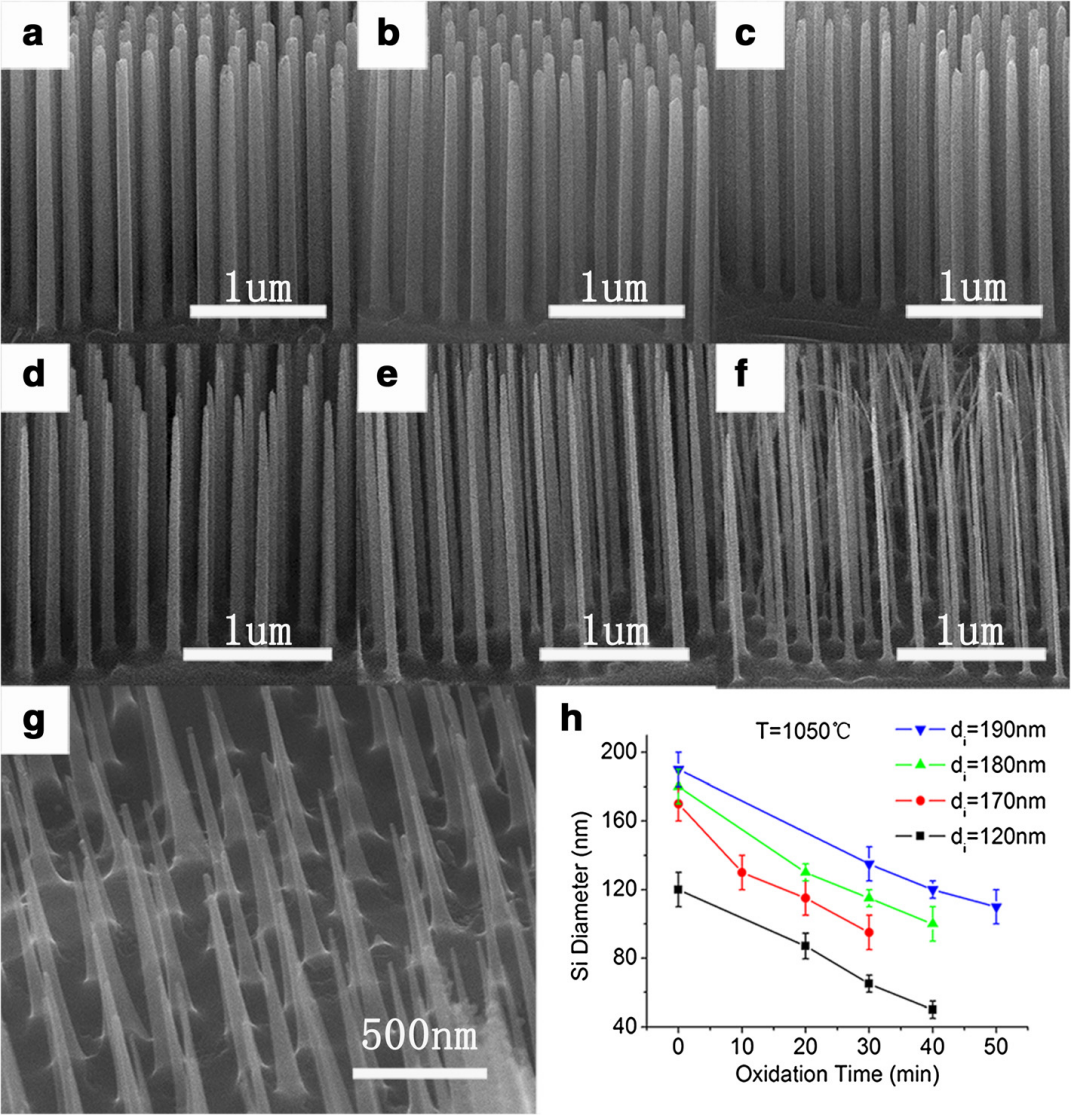
**SEM images of samples after dry oxidation. (a)** to **(f)** SEM images of samples after first-step oxidation of **(a)** 20, **(b)** 30, and **(c)** 40 min, and two-step oxidation of **(d)** 30 + 20 min, **(e)** 30 + 30 min, and **(f)** 30 + 40 min. **(g)** SEM image for the sample with reduced diameter of around 50 nm only by one-step oxidation. **(h)** The silicon diameter and oxidation time relationship for samples with typical initial diameters.

The self-limiting effect can take place only when the diameter of the SiNWs is around 50 nm. Dry oxidation and post-chemical etching were carried out to reduce the SiNW diameter to this dimension. It is found that the oxidation at 1,070°C for 1 h could reduce the diameter of the SiNWs down to around 50 nm, while the diameter along the nanowires became inhomogeneous, indicating an axially inhomogeneous oxidation rate during the oxidation process. A two-step oxidation was employed here, in which the oxidation was terminated, and the formed oxide was removed before the inhomogeneous oxidation rate took place. Figure 5a,b,c shows the SiNWs after first-step oxidation at 1,050°C and post-chemical etching, the initial diameter of which is about 175 nm. The dimension of the residual nanowires was about 133, 118, and 104 nm when the first-step oxidation lasted for 20, 30, and 40 min, respectively. It is found that the diameter along the nanowires is almost uniform, with little difference from the morphology induced by the Ag-assisted chemical etching. The samples with diameter of approximately 118 nm were chosen for the second-step oxidation, and the results were listed in Figure 5d,e,f. The diameter was further reduced to about 77, 61, and 48 nm when the oxidation time was 20, 30, and 40 min, respectively. It is determined that for the sample with initial diameter of about 175 nm, dry oxidation with '30 + 40 min’ is available to obtain SiNWs proper for the future self-limiting oxidation.

As a fabrication method with so many steps, especially with the RIE step which fluctuates a lot, it is hard to obtain nanowire arrays of equal diameter for dry oxidation from every sample. This instability can be corrected by dry oxidation treatment. For each 3 cm × 3 cm silicon substrate, several 2 mm × 5 mm pieces would be cut down prior to the formal experiment to try out the proper oxidation time parameters through the abovementioned methods. Then, the tried-out parameters would be applied to the whole remaining sample. Figure 5h summarizes the dependence of the reduced diameter of the SiNWs on the oxidation time for samples with typical initial diameters.

**Figure 6:**
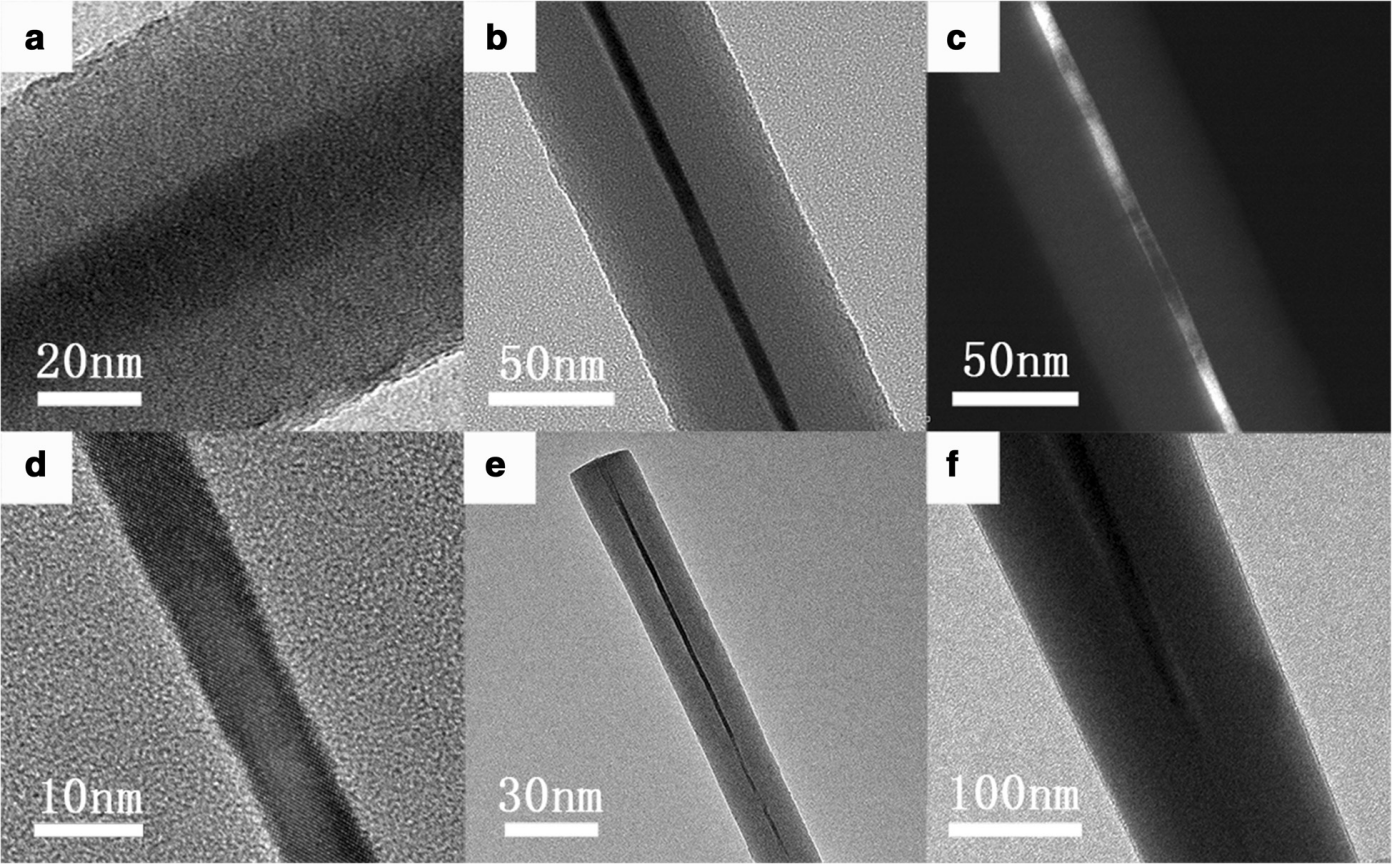
**TEM images of samples after self-limiting oxidation. (a)** to **(f)** TEM images of samples after 10-h self-limiting oxidation at **(a)** 750°C, **(b)** to **(e)** 800°C, and **(f)** 850°C.

Figure 6 displays the TEM images of SiNWs after 10-h self-limiting oxidation at different temperatures. Due to the insertion of oxygen atoms, the total diameter of SiNWs expanded to approximately 80 nm. Molecular dynamics simulation reveals that since the oxidation process is strongly suppressed by the huge compressive stress which is concentrated in the oxide region near the SiO_2_/Si interface, the oxidation process will be self-terminated, and a core-shell structure is formed instead of being completely oxidized [30]. A core diameter of about 20 nm was obtained from the sample oxidized at 750°C (Figure 6a). When the oxidation temperature was enhanced to 800°C, the core diameter could be reduced to around 7 nm, as shown in Figure 6b. Dark field image (Figure 6c) and high-resolution transmission electron microscopy (HRTEM) image (Figure 6d) further demonstrate that the core-shell structure is made up of a single crystal core and an amorphous shell. In addition, the homogeneous core diameter can be confirmed by the low magnification image (Figure 6e), which is around 6 nm at the top and approximately 9 nm at the bottom. For the oxidation conducted at 850°C, most SiNWs were completely oxidized, and there were residual silicon cores only at the root of some nanowires with outside diameters larger than 150 nm, as presented in Figure 6f.

## Conclusions

In summary, this study illustrates a promising technique of preparing controllable single crystal SiNW arrays covering a large area. PS monolayer template was employed to prepare the nanoporous Ag film as catalyzer for the solution etching process, which would yield SiNW arrays. Two-step dry oxidation at 1,050°C reduced the nanowire diameter to around 50 nm while preventing nanowires from becoming sharp. Temperature is crucial for the self-limiting oxidation process. After oxidation at 800°C, the inner diameter of the core-shell SiNW arrays can be controlled below 10 nm within a tight tolerance. The fabrication process is easy to conduct and has good reproducibility. As the experiment was conducted top-down on single crystal silicon wafers, the SiNWs produced through this way have low defect concentration and consistent crystallography orientation. In addition, the core-shell structure guarantees their property stability in atmosphere. Since this technique combines functionality and economy, it is of high possibility to be applied to silicon-based optical devices in the future.

## Abbreviations

EBL: Electron beam lithography; FE-SEM: Field-emission scanning electron microscope; HRTEM: High-resolution transmission electron microscopy; PS: Polystyrene; RIE: Reactive ion etching; SDS: Sodium dodecyl sulfate; SEM: Scanning electron microscopy; SiNW: Silicon nanowire; TEM: Transmission electron microscopy; VLS: Vapor–liquid-solid.

## Competing interests

The authors declare that they have no competing interests.

## Authors’ contributions

SS carried out the fabrication and characterization of the study and drafted the manuscript. LL conceived of the study, participated in its design and preparation, analyzed the results, and helped draft the manuscript. JF participated in the design of the study and helped draft the manuscript. ZL and ZZ participated in the design and coordination of the study. All authors read and approved the final manuscript.

## Authors’ information

All authors belong to School of Materials Science and Engineering, Tsinghua University, People's Republic of China. SS is a master candidate interested in silicon-based light emission. LL is a Ph.D. candidate concentrating on semiconductor nanomaterials. ZL is an associate professor whose research fields include thin film material and nuclear material. JF is a professor working on thin film material and nanomaterials. ZZ is the school dean professor with research interest in nanostructures and SERS effect.
